# Resveratrol ameliorates atrazine-induced caspase-dependent apoptosis and fibrosis in the testis of adult albino rats

**DOI:** 10.1038/s41598-024-67636-z

**Published:** 2024-07-31

**Authors:** Hala Mohamed Hassanin, Asmaa A. Kamal, Omnia I. Ismail

**Affiliations:** 1 Human Anatomy and Embryology Department, Faculty of Medicine, Assiut University, Assiut, 71515 Egypt; 2 Medical Biochemistry Department, Faculty of Medicine, Assiut University, Assiut, Egypt

**Keywords:** Atrazine, Resveratrol, Testis, qPCR, Caspase 3, iNOS, Biochemistry, Cell biology, Health care, Medical research, Molecular medicine

## Abstract

Pesticides like atrazine which are frequently present in everyday surroundings, have adverse impacts on human health and may contribute to male infertility. The work aimed to analyze the histological and biochemical effects of atrazine on the testis in adult albino rats and whether co-administration with resveratrol could reverse the effect of atrazine. Forty adult male albino rats in good health participated in this study. They were categorized at random into four groups: the Group Ӏ received water through a gastric tube for two months every day, the Group ӀӀ received resveratrol (20 mg/kg body weight (b.w.)) through a gastric tube for two months every day, the Group ӀӀӀ received atrazine (50 mg/kg bw) through a gastric tube for two months every day, the Group ӀV received concomitant doses of atrazine and resveratrol for two months every day. The testes of the animals were then carefully removed and prepared for biochemical, immunohistochemical, light, and electron microscopic studies. Atrazine exposure led to a significant decrease in serum testosterone hormone level, upregulation of caspase 3 and iNOS mRNA levels, destructed seminiferous tubules with few sperms in their lumens, many collagen fibres accumulation in the tunica albuginea and the interstitium, abnormal morphology of some sperms as well as many vacuolations, and damaged mitochondria in the cytoplasm of many germ cells. Concomitant administration of resveratrol can improve these adverse effects. It was concluded that atrazine exposure is toxic to the testis and impairs male fertility in adult rat and coadministration of resveratrol guards against this toxicity.

## Introduction

Atrazine is one type of herbicide, which is frequently employed in order to weed dry crops for example sorghum, corn, and trees of fruit^1^. It selectively inhibits the photosynthesis of grassland and broadleaf weeds^2^.

The maximum permitted concentration (MAC) of atrazine in water is 5 μg/L, suggesting it is a harmful herbicide^3^. The concentration of atrazine in the soil is different from site to site. There may be a connection between the increased pesticide use in farmlands and the higher atrazine levels in some locations^4^.

Atrazine is a persistent substance that affects crops after it is removed from the soil. It can stay in clay loam soil for several years at temperatures below 20 °C^5^.

Atrazine has a 2–4 week half-life, although its metabolites and residuals can be found for up to 4 months following application. The subsurface is where atrazine penetrates and gets adsorbed to soil grains, where its rate of disintegration is considerably slowed down^6^. The aquatic environment receives between 1 and 6% of the atrazine administered^7^.

Exposure to soil contaminants by atrazine can occur through three primary routes: first, the respiratory system is exposed to contaminated particles through breathing. The second is the skin's dermal absorption of particles that have attached. Third, ingesting dirt particles directly^8^.

Moreover, atrazine has contaminated a variety of ecosystems due to its low vapor pressure, long half-life in soil, and great mobility. The US Environmental Protection Agency has classified atrazine toxicity as class III; yet, given the possibility of groundwater pollution, its significance cannot be discounted^9^.

Several research on the toxicological profile of atrazine mentioned that its exposure potentially endangers human and environmental health. There have been many reports of atrazine's harmful effects on several systems in humans, but unfortunately, the bulk of these scientific findings focused on animal studies^10^.

The reproductive and endocrine systems are the main targets of atrazine poisoning. It has been demonstrated that the endocrine-disrupting chemical atrazine has an impact on the behavior, development, and reproduction of several species^11–13^. Increasing evidence suggests that it may be harmful to both male and female reproductive systems^14^.

In adult females, atrazine consumption has been linked to early onset of pituitary and mammary cancers, extension of the estrous cycle, decreased weight gain caused by estradiol in the uterus, reduced uterine cytosolic progesterone receptor binding, and reduced estradiol-caused uterine weight growth^15,16^. Male adults who are exposed to atrazine may experience reduced weights in the anterior pituitary, the prostate, and the hypothalamus, decreased levels of dihydrotestosterone attaching to the androgen receptor, as well as decreased spermatozoa quantity and motility^17^.

One of the most significant health issues that affects couples is infertility. Around 30% of these cases are caused by male factors. There are other factors including chemotherapy, environmental toxins, and drug use that can harm spermatogenesis and affect normal sperm production^18^.

Pesticides and endocrine disruptors like atrazine, which are frequently present in daily life surroundings and are increasingly being investigated for their impact on human health, may have contributed to male infertility. The noted drop in sperm concentration may have also been strongly influenced by these factors^2^.

Resveratrol (3,5,4-trihydroxystilbene RSV) is a naturally occurring phytoalexin antioxidant present in many plants, such as peanuts, blueberries, and grapes. The biological actions of RSV, which include anti-inflammatory, cytoprotective, and anti-aging properties, have garnered increased attention^19^. Red wine and grapes are the main sources of RSV, a chemical that has positive effects on the reproductive system^20^, so we thought that it shows promise in treating atrazine-induced reproductive toxicity.

RSV has been recognized as an effective treatment for preventing male reproductive impairments brought on by diabetes mellitus by reducing oxidative stress-mediated inflammation of the testicles in rats^21^.

There is proof that consuming resveratrol considerably decreased the levels of malondialdehyde and increased the mRNA levels of the antioxidant enzyme testicular superoxide dismutase^22^.

The basic mechanism underlying the RSV's beneficial effects on health is thought to be its antioxidant impact. Additionally, research on RSV demonstrated that it has powerful therapeutic effects against several illnesses like respiratory viral infections, diabetes mellitus, Alzheimer's disease, and kidney disorders^23^. Resveratrol is a power antioxidant protecting the structure of testis against several chemicals like methotrexate^24^, cisplatin^25^, monosodium glutamate^26^ and isoflurane^27^. Thus, we hypothesis that resveratrol can ameliorate atrazine induced testicular damage and we hope to introduce it as a novel agent against atrazine toxicity in the testis.

The main reason for selecting atrazine is based on many facts such as its extensive usage, extended half-life in the environment, and its endocrine disruptor ability. Moreover, the impact of resveratrol on the atrazine-mediated testicular damage is uncertain. The present study was conducted to analyze the histological and biochemical effects of atrazine on the testis in adult albino rats and whether co-administration with resveratrol could reverse the effect of atrazine.

## Materials and methods

### Chemicals

Atrazine and resveratrol were bought from Sigma-Aldrich Co., St. Louis, MO, USA.

### Animals

Forty adult three-month-aged male albino rats in good health participated in this study. They weighed between 200 and 250 g. The rats were acquired from the Animal House of Faculty of Medicine, Assiut University. They were then kept in carefully regulated laboratory settings, including a 12-h light and 12-h dark cycle at 25 °C, a regular rodent pellet meal, and unlimited access to water.

The method of power analysis was used to calculate sample size utilizing software G Power as described by Charan & Kantharia^28^.

The animals were categorized at random into four groups (ten rats in each group):

- The Group Ӏ (negative control group): received water through a gastric tube for two months every day.
- The Group ӀӀ (positive control group): received resveratrol (20 mg/kg body weight (b.w.)) through a gastric tube for two months every day according to^29^.
- The Group ӀӀӀ (atrazine-treated group): received atrazine (50 mg/kg bw) through a gastric tube for two months every day according to^30^.
- The Group ӀV (atrazine + resveratrol treated group): received concomitant daily doses of atrazine and resveratrol as in the previously outlined regimen.

24 h after the last treatment, half of the experimental male rats in each group (5 rats in each group) were mated with healthy females. For two estrus cycles, a male and two female rats were kept together. The impact of atrazine on the fertility capacity was assessed.

After fertility capacity evaluated, the animals were sacrificed under anesthesia by intraperitoneal injection of 25 mg/kg sodium thiopental. Blood samples were taken from the eye sinus on EDTA tubes. The plasma was then extracted within 30 min of collection by centrifugation for 15 min at 3000 rpm, and it was stored at minus 20 °C to measure plasma testosterone. The testes of the animals were carefully removed and prepared for biochemical, immunohistochemical, light, and electron microscopic studies.

### Ethics declaration

The practices for animal handling and experimental methods strictly adhered to CPCSEA guidelines and the ARRIVE guidelines^31,32^. Ethics permission was received from the Ethics Committee at Faculty of Medicine, Assiut University in Egypt (IRB local approval number: 17300976).

### Fertility study

For the fertility test, a total of forty prestrous fertile untreated female rats were employed. A male rat and two untreated female rats lived together for two estrus cycles. It was determined that the existence of a vaginal plug marked the beginning of the pregnancy and served as an indicator of a successful mating^33^.

The following formula was used to determine a fertility test^34^:

$$ {\text{Fertility}}\;{\text{Success }}\left( \% \right) = \frac{{{\text{Pregnancy}}\;{\text{Females}} \times 100 }}{{{\text{Mated}}\;{\text{females}}}} $$

### Real‐time quantitative polymerase chain reaction (qPCR)

Thermo Scientific Gene JET RNA purification kit (catalog no. #K0731; Thermo Scientific) was used to extract total RNA. The purity and concentration of RNA fraction were determined by using Nanodrop® (Epoch Microplate Spectrophotometer; Biotech). Before being used, RNA samples were kept at − 80 °C. Using the Thermo Scientific High-Capacity cDNA Reverse Transcription kit (catalog no.4368813), complementary DNA (cDNA) was created. The cDNA was kept cold at − 20 °C until the next step was taken. The Maxima SYBR Green qPCR master mix (2 ×) kit (Catalog no. K0251, Thermo Scientific) was utilized for the qPCR process. RT-qPCR was conducted using the Applied Biosystems 7500 Fast Real-time PCR equipment (Applied Biosystems) with beta-actin serving as the internal control. Following optimization, these were the PCR cycle conditions: first denaturation stage of 95 °C for 1 cycle of 7 min, then 40 cycles of 20 s at 95 °C and 50 s at 60 °C. The relative transcription levels of mRNA were calculated using the equation of fold change 2 − ΔΔCT method. Table 1 lists the primers used. All primers were synthesized by Thermo Fisher Scientific (USA).

**Table 1:** Primers used for Rt pcr.

	Forward	Reverse	Accession number
iNOS	GAGTGGCAACATCAGGTC	GGTCTCGGACTCCAATCT	NM_012611 XM_220732
Casp-3	TCTTCAGAGGCGACTACTGC	TCCGGTTAACACGAGT GAGG	NM_012922
B-actin	GATTACTGCCCTGGCTCCTAGC	CTCCTGCTTGCTGATCCACATC	NM_031144.3

### Estimation of testosterone hormone level

Testosterone hormone level in plasma was measured by an enzyme‐linked immunoassay (ELISA) Kit (catalog no. E-EL-0155) supplied by Elabscience.

### Light microscopic study

The right testis from all animals was prepared for light microscopic examination after being preserved in 10% neutral buffered formalin. For a standard histological investigation, the paraffin section (4–5 μm) were produced and stained with Hematoxylin and Eosin (H&E). Also, a picrosirius red staining procedure was used to reveal the distribution of the collagen fibers^35^. The following semiquantitative grade was applied to testicular lesions: 0 represents normal, undamaged histoarchitecture; 1 denotes mild lesions; 2 denotes moderate lesions; and 3 denotes severe damage^36^.

### Transmission electron microscopic study (TEM)

The left testes fragments from the mid-anterior area (about 3 mm^3^) were preserved using a solution containing 2.5 percent glutaraldehyde and 2 percent paraformaldehyde for a whole day at 4 °C. The samples were then washed with the cacodylate buffer twice in 20 min. The fixation using 1 percent osmium tetroxide was done for 120 min at 4 °C. After being dehydrated in increasing grades of ethyl alcohol, the specimens were submerged for 15 min in a solution containing equal amounts of ethanol and acetone, and then for an additional 15 min in acetone. After that, the specimens were immersed in equal amounts of epon and acetone for an hour, followed by a one-hour immersion in a 25:50 acetone: epon mixture, and finally, they were submerged in epon exclusively in embedding capsules and left overnight in an oven set at 60 °C for polymerization. Semithin section (1 μm) were prepared and stained with a 1 percent solution of toluidine blue^37^. Ultrathin section (60 nm) were prepared and stained with lead citrate and uranyl acetate for analysis by (TEM JEOL JEM- 100 SX) in the Electron Microscopy Unit, Assiut University. The following semiquantitative grade was applied to testicular lesions: 0 represents normal, undamaged histoarchitecture; 1 denotes mild lesions; 2 denotes moderate lesions; and 3 denotes severe damage^36^.

### Scanning electron microscopy (SEM)

The left testes fragments from the mid-anterior area (about 3 mm^3^) were fixed in Karnovsky's fixative after whole body perfusion fixation, then they were put three times in the wash with 0.1 M sodium phosphate buffer (pH 7.2), and finally in rising saccharose solutions (0.5, 1.5, 3 percent). Before being subjected to critical point drying, the specimens were first frozen in liquid nitrogen, broken, post-fixed in one percent osmium tetroxide, bathed, and then dehydrated in gradually increasing ethanol concentrations. The samples were put on stubs, given a gold sputter coating, and examined under the scanning electron microscope (Geol–JMS 560) in the Electron Microscopy Unit, Assiut University^38^. The following semiquantitative grade was applied to testicular lesions: 0 represents normal, undamaged histoarchitecture; 1 denotes mild lesions; 2 denotes moderate lesions; and 3 denotes severe damage^36^.

### Immunohistochemical study

The testicular sections with a thickness of 3 microns were hydrated and deparaffinized. To bring out the antigen, sections were treated with citrate buffer for approximately ten minutes. In order to reduce endogenous peroxidase activity, the sections underwent treatment with 0.3 percent H2O2 for 30 min. Afterward, the two primary antibodies were incubated on separate slides: polyclonal rabbit caspase-3 (dilution 1:150, rabbit, Catalog# RB-1197-B Thermo Fisher Scientific, CA, USA) and rabbit inducible nitric oxide synthase (iNOS) (dilution 1:150, rabbit, Catalog#MA5-17,139 Thermo Fisher Scientific, CA, USA) for one night at room temperature in humidity. The next day, the sections were treated for two hours using the anti-goat biotinylated secondary antibody (dilution 1:100, Abways) at room temperature. For every immunostaining, there were both positive and negative controls carried out without primary antibodies. Then, the sections underwent staining using the Beyotime Biotechnology Co., LTD. diaminobenzidine chromogen kit, followed by hematoxylin counterstaining, dehydration, xylene clearing, and covering^39^.

### Morphometric analysis

The histomorphometric assessment of the semithin sections at a magnification of × 40 was done to measure the height of germinal epithelium, and the area of seminiferous tubules for each sample using computerized image analyzer system software (Leica Q 500 MCO; Leica, Wetzlar, Germany) connected to a camera joined to a Leica universal microscope at Human Anatomy and Embryology Department, Faculty of Medicine, Assiut University, Assiut –Egypt. The area percent of collagen fibres in Picrosirius red-stained sections and area percent of caspase 3 and iNOS positive expression in the immunostained sections were calculated using the Image J software's cell counter plugin (version 1.52, Public Domain) in twenty randomly selected, non-overlapping fields from every animal in every group at a magnification of × 400.

### Statistical analysis

The results are expressed as the mean ± standard deviation of the mean (SD) and analyzed by one-way analysis of variance (ANOVA) followed by Tukey's post hoc test. A p-value of less than 0.05 indicated that there was a significant difference between the groups.

### Ethics approval and consent to participate

Ethical approval was obtained from the Ethical Committee of the Faculty of Medicine, Assiut University, Egypt. All methods were performed in accordance with the relevant guidelines and regulations and in compliance with ARRIVE guidelines for the care and use of experimental animals by the committee for supervision of Experiment on animals (CPCSEA) and the National Institute of Health NIH Protocol. Consent to participate from participants is not applicable as it is an animal experimental study.

## Results

### Effect of resveratrol and atrazine treatment on male fertility capacity

The ability of male rats given atrazine to fertilize was significantly decreased compared to the control rats. An insignificant increase in number of pregnant rats and fertility success in females mated with rats co-administered with atrazine and resveratrol compared to the atrazine-treated group (Table 2).

**Table 2:** The comparison of number of pregnant rats and the fertility success (%).

Groups	Total number of pregnant rats	The fertility success
Group I	9	90% ± 22.36
Group II	10	100% ± 0.0
Group III	4	40% ± 41.83^a,b^
Group IV	7	70% ± 27.39
*P*-value	–	0.0158*

Data are represented as Mean ± SD. * means statistically significant difference.

^a^statistically significant as compared with the group I, *P* < 0.05.

^b^statistically significant as compared with the group II, *P* < 0.05.

### Effect of Resveratrol and atrazine treatment on the serum testosterone level

**Figure 1:**
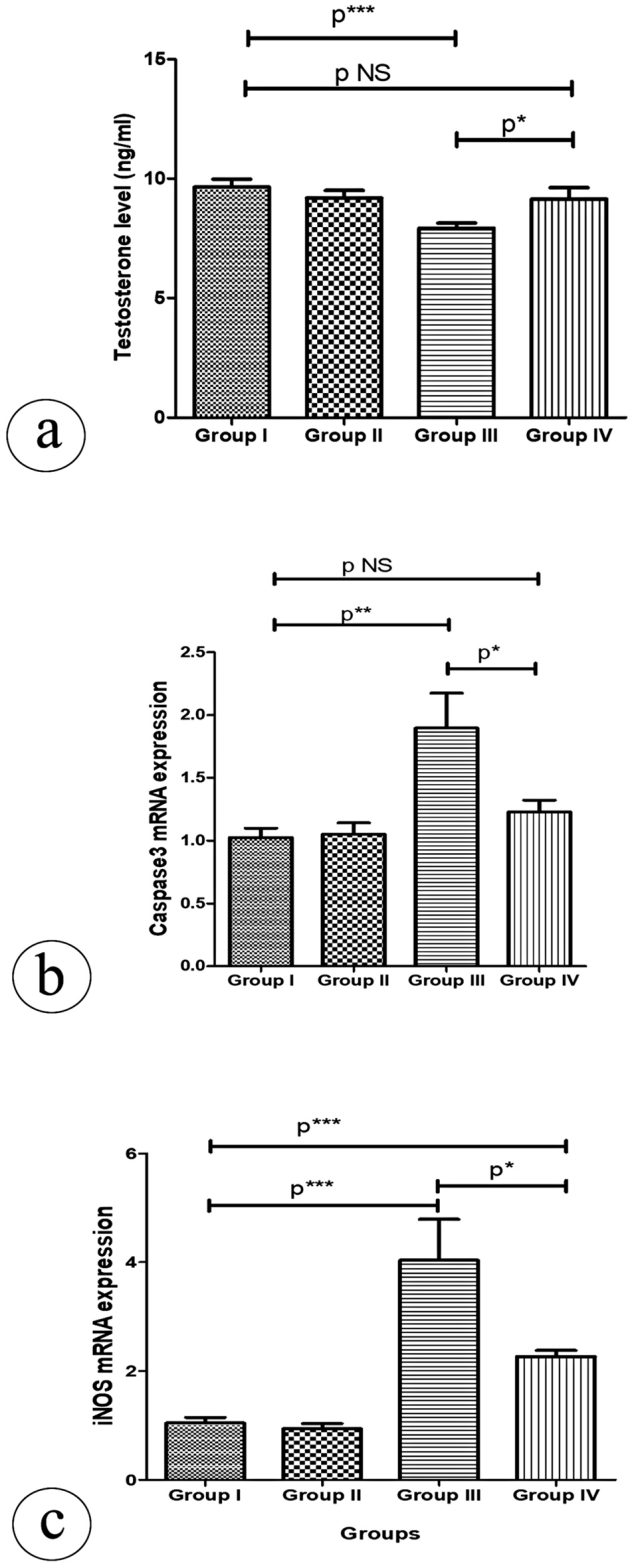
(**a**) Plasma testosterone level (ng/ml) in all studied groups. Values are taken from 10 mice in each group. (**b**) Relative quantitative expression of mRNA level of caspase 3 in testicular tissue of different study groups. (**c**) Relative quantitative expression of mRNA level of iNOS in testicular tissue of different study groups. Both genes expression levels were normalized to beta-actin. Data are expressed as means ± SD. p* (< 0.05). p*(< 0.01). p**(< 0.001) .p NS (non-significant > 0.05).

There was a notable drop in the mean serum level of testosterone hormone in the atrazine-treated group compared to the control group. In contrast, resveratrol co-treatment led to an increase in the mean serum testosterone hormone level compared to the atrazine-treated group (Fig. 1a). The intra-assay coefficient variation (CV) is 14.65%, while the inter-assay CV is 12.46% for serum testosterone hormone level.

### Effect of resveratrol and atrazine treatment on the caspase 3 and iNOS mRNA level

The present data showed that the caspase 3 mRNA level was significantly upregulated in the atrazine-treated group compared to both control groups. In the atrazine + resveratrol-treated group; the caspase 3 mRNA level was significantly decreased in comparison to the atrazine-treated group but there was no significant difference compared to both control groups (Fig. 1b). The intra-assay CV of Ct values is 5.11%, while the inter-assay CV is 4.26% for caspase 3 expression.

The expression of iNOS mRNA was significantly higher in the testicular tissue of the atrazine-treated group in comparison to both control groups. However, its expression level was significantly decreased in the atrazine + resveratrol-treated group compared to the atrazine-treated group, but still significantly high in comparison to both control groups (Fig. 1c). The intra-assay CV of Ct values is 4.56%, while the inter-assay coefficient variation is 3.39% for iNOS expression.

### Resveratrol attenuated histopathological alterations in the testes of the atrazine-intoxicated rats

The histological structure of the testicular sections from the positive and negative control groups was more or less identical, according to examination. Consequently, the results listed under "Control Group" were representative of both control groups.

**Figure 2:**
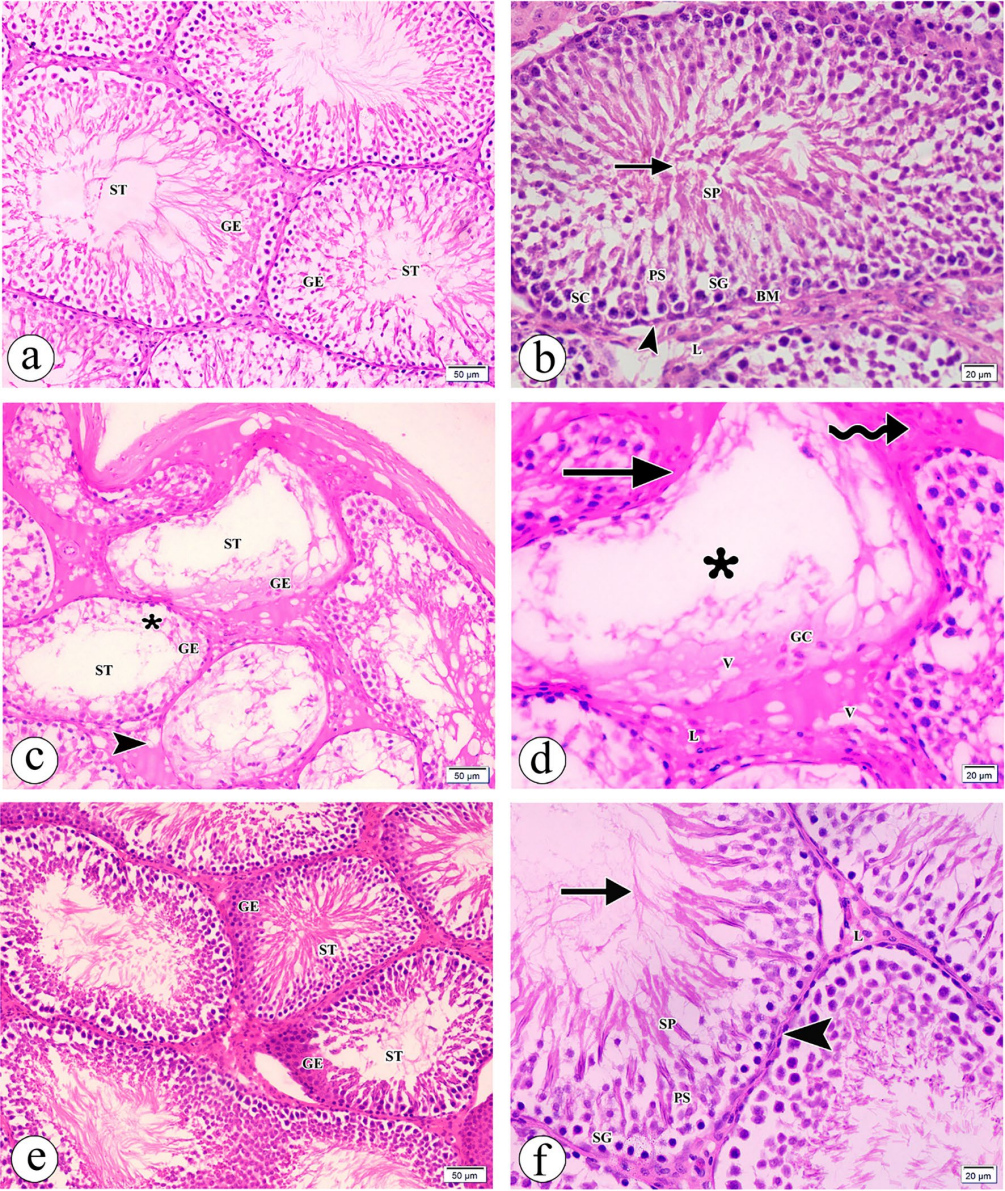
Photomicrographs of the sections of the testes in (**a**) the control group showing uniform seminiferous tubules (ST) having germinal epithelium (GE) lining them at various stages of maturity. (**b**) The control group showing a seminiferous tubule has spermatogonia (SG) appearing as small dark cells located extremely close to the basement membrane (BM) having fattened nucleus of the myoid cell (arrowhead), Sertoli cells (SC) with dense nuclei, primary spermatocytes (PS) with a central large nucleus, and spermatids (SP) with pale cytoplasm. The Leydig cells (L) in the interstitial spaces and crowded sperm flagella (arrow) in the lumen are noticed. (**c**) The atrazine-treated group showing destructed seminiferous tubules (ST) having damaged germinal epithelium (GE) separating from the basement membrane (asterisk). Wide vacuolated interstitial spaces (arrowhead) are seen. (**d**) The atrazine-treated group showing a seminiferous tubule having few germinal cells (GC), irregular basement membrane (arrow), absent sperms in the lumen (asterisk), and many vacuolations (V). Few Leydig cells (L) and acidophilic material deposition (wavy arrow) in the interstitial spaces are detected. (**e**) The atrazine + resveratrol-treated group showing restoration of normal structure of seminiferous tubules (ST) and germinal epithelium (GE). (**f**) the atrazine + resveratrol-treated group showing a seminiferous tubule with normal-appearing spermatogonia (SG), primary spermatocytes (PS), spermatids (SP), crowded sperm flagella (arrow) in the lumen and the basement membrane (BM) having fattened nucleus of myoid cell (arrowhead). The Leydig cells (L) in the interstitial spaces are observed. (**H** & **E** a,c,e × 200, Scale bar = 50 μm & b,d,f × 400, Scale bar = 20 μm).

When testicular tissue from the control group was examined under a light microscope, it was discovered normal structure of mature functioning homogeneous, seminiferous tubules lined with germinal epithelium at different stages of development (Fig. 2a). The seminiferous tubules had spermatogonia appearing as small dark cells located extremely close to the basement membrane having fattened nucleus of myoid cell, Sertoli cells, primary spermatocytes with central large nucleus, and spermatids with pale cytoplasm. The Leydig cells in the interstitial spaces and crowded sperm flagella in the lumen were noticed (Fig. 2b). On the other hand, atrazine treatment led to destructed seminiferous tubules and damaged germinal epithelium separating it from the basement membranes. The blood vessels became dilated and congested. Wide vacuolated interstitial spaces were seen (Fig. 2c). Additionally, the seminiferous tubules had few germinal cells, irregular basement membranes, absent sperms in the lumen, and many vacuolations. Few deeply stained Leydig cells and acidophilic material deposition in the interstitial spaces were detected (Fig. 2d). Surprisingly, resveratrol concomitant administration provoked restoration of normal structure of some seminiferous tubules and germinal epithelium (Fig. 2e). The seminiferous tubules lined with normal appearing spermatogonia, primary spermatocytes, spermatids, and crowded sperm flagella appeared in the lumen. The basement membranes formed of fattened nuclei of myoid cells. The Leydig cells in the interstitial spaces were observed (Fig. 2f).

**Figure 3:**
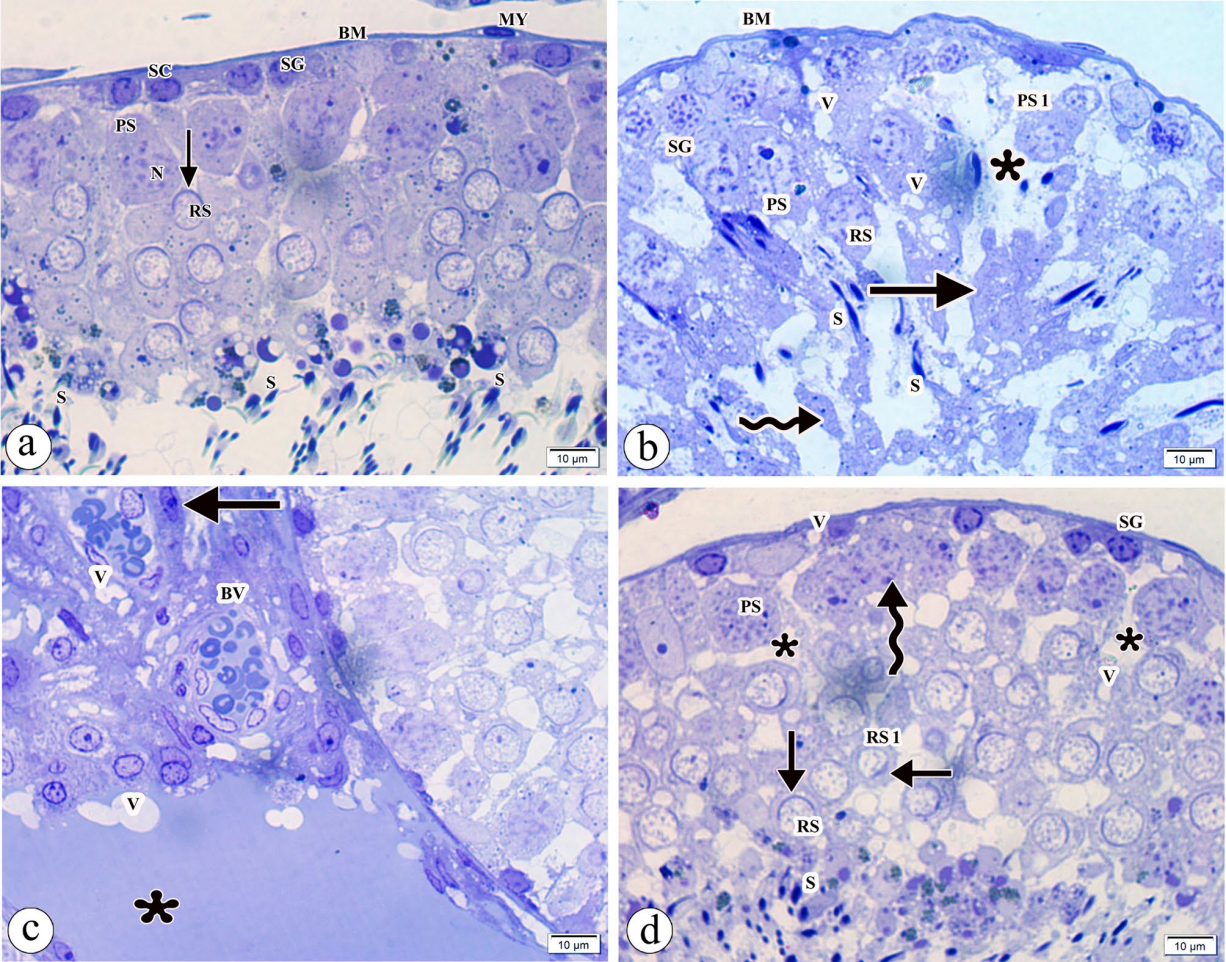
Photomicrographs of the semithin sections of the testes in (**a**) the control group showing the germinal epithelium consists of the spermatogonia (SG) appearing as small dark cells, the primary spermatocyte (PS) having large nucleus (N) with condensed chromosomes, the round spermatid (RS) with acrosomal cap (arrow) and many sperms (S) with well-formed head and tail. The basement membrane (BM), myoid cells (MY), and the Sertoli cell (SC) are observed. (**b**) The atrazine-treated group showing damaged germinal epithelium with the spermatogonia (SG) positioned away from the wavy basement membrane (BM), the irregular outline of the nucleus of primary spermatocyte (PS), shrunken nucleus of primary spermatocyte (PS1), and round spermatid (RS) having loss of acrosomal cap. Some ill-defined germ cells (arrow) are seen. Other cells shed and slough to the lumen (wavy arrow). Many vacuolations (V), wide space (asterisk) between the germ cells, and few sperms (S) are noticed. (**c**) The atrazine-treated group showing many vacuolations (V), deeply stained Leydig cells (arrow), dilated congested blood vessels (BV), and exudate (asterisk) in wide interstitial space. (d) the atrazine + resveratrol-treated group showing partially improving germinal epithelium with an irregular outline of primary spermatocyte (PS), some round spermatids (RS) appear with acrosomal cap (arrow), but other round spermatids (RS) have partial loss of acrosomal caps (arrow). Some primary spermatocytes have ill-defined cell borders (wavy arrow). Many vacuolations (V), wide space (asterisk) between the germ cells, and sperms (S) are seen. (Toluidine blue × 1000, Scale bar = 10 μm).

Examination of the semithin sections of the testes in the control group demonstrated normal cytoarchitecture of the germinal epithelium (Fig. 3a). On the contrary, the atrazine-treated group showed damaged germinal epithelium with the spermatogonia positioned away from wavy basement membrane, irregular outlines of nuclei of primary spermatocytes, shrunken nuclei of other primary spermatocytes, and round spermatids with loss of acrosomal cap. Some ill-defined germ cells were seen. Other cells shed and sloughed to the lumen. Many vacuolations, wide space between the germ cells, and few sperms were noticed. Deeply stained Leydig cells, dilated congested blood vessels, and exudate in wide vacuolated interstitial space were detected (Fig. 3b,c). Interestingly, the atrazine + resveratrol treated group showed partially improving germinal epithelium with many sperms, but irregular outlines of primary spermatocytes were observed. Some primary spermatocytes had ill-defined cell borders. Some round spermatids appeared normal with acrosomal cap, but other round spermatids revealed partial loss of acrosomal cap. Many vacuolations and wide space between the germ cells were still present (Fig. 3d). Table 3 displays the histopathological scoring observed in the different groups.

**Table 3:** Histopathological scoring under light microscopic examination, scanning electron microscopic examination and transmission electron microscopic examination.

Lesion	Group I	Group II	Group III	Group IV	*P*-value
Under light microscopic examination					
Destructed seminiferous tubules	0.20 ± 0.42	0.30 ± 0.48	2.30 ± 0.48^a,b,d^	1.20 ± 0.57^a,b,c^	< 0.0001*
Damaged germinal epithelium	0.30 ± 0.48	0.20 ± 0.42	2.10 ± 0.74^a,b,d^	1.30 ± 0.67^a,b,c^	< 0.0001*
Dilated congested blood vessels	0.40 ± 0.51	0.60 ± 0.50	2.60 ± 0.52^a,b,d^	1.60 ± 0.7 ^a,b,c^	< 0.0001*
Wide vacuolated interstitial spaces	0.40 ± 0.51	0.41 ± 0.52	2.30 ± 0.67^a,b,d^	1.50 ± 0.53 ^a,b,c^	< 0.0001*
Irregular basement membranes	0.40 ± 0.52	0.30 ± 0.48	1.80 ± 0.79^a,b,d^	1.00 ± 0.67^c^	< 0.0001*
Absent sperms in the lumen	0.30 ± 0.48	0.10 ± 0.32	2.40 ± 0.52^a,b,d^	1.40 ± 0.70^a,b,c^	< 0.0001*
Vacuolations	0.40 ± 0.52	0.20 ± 0.42	2.20 ± 0.63^a,b,d^	0.80 ± 0.42 ^c^	< 0.0001*
Deeply stained Leydig cells	0.30 ± 0.48	0.30 ± 0.49	2.20 ± 0.62^a,b,d^	0.80 ± 0.63 ^c^	< 0.0001*
Acidophilic material deposition in the interstitial spaces	0.10 ± 0.32	0.0 ± 0.0	1.30 ± 0.67^a,b,d^	0.30 ± 0.84^c^	< 0.0001*
Under scanning electron microscopic examination					
Shrunken seminiferous tubules with irregular outlines	0.40 ± 0.55	0.20 ± 0.45	2.40 ± 0.90^a,b,d^	1.20 ± 0.48^c^	< 0.0001*
Separation of the germinal epithelium from the basement membrane	0.20 ± 0.45	0.20 ± 0.45	2.60 ± 0.55^a,b,d^	1.20 ± 0.84^c^	< 0.0001*
Sperms with detached parts of the heads	0.40 ± 0.55	0.20 ± 0.45	2.20 ± 0.48^a,b^	1.20 ± 0.45	0.0002*
Sperms with tail splitting	0.40 ± 0.52	0.40 ± 0.54	2.40 ± 0.55^a,b,d^	1.40 ± 0.55^a,b,c^	< 0.0001*
Abnormal morphology of the heads of sperms	0.60 ± 0.55	0.40 ± 0.55	2.20 ± 0.83^a,b,d^	1.60 ± 0.53^b^	0.0010*
The membrane disruptions in the sperms	0.40 ± 0.55	0.20 ± 40	2.20 ± 84^a,b,d^	1.20 ± 83	0.0012*
Cytoplasmic droplets throughout the tail of sperms	0.20 ± 0.45	0.20 ± 0.44	1.80 ± 0.43^a,b,d^	1.00 ± 0.70	0.0004*
Under transmission electron microscopic examination					
Damaged Sertoli cells	0.20 ± 0.44	0.0 ± 0.0	2.00 ± 0.72^a,b,d^	1.00 ± 0.82	0.0002*
Damaged primary spermatocytes	0.60 ± 0.54	0.60 ± 0.52	2.20 ± 0.83^a,b,d^	1.40 ± 0.57	0.0026*
Damaged rounded spermatid	0.20 ± 0.44	0.20 ± 0.45	2.20 ± 0.83^a,b,d^	1.20 ± 0.84	0.0005*

Data are represented as Mean ± SD. * means statistically significant difference.

^a^statistically significant as compared with the group I, *P* < 0.05.

^b^statistically significant as compared with the group II, *P* < 0.05.

^c^statistically significant as compared with the group III, *P* < 0.05.

^d^statistically significant as compared with the group IV, *P* < 0.05.

### Effect of resveratrol on atrazine-induced testicular fibrosis

**Figure 4:**
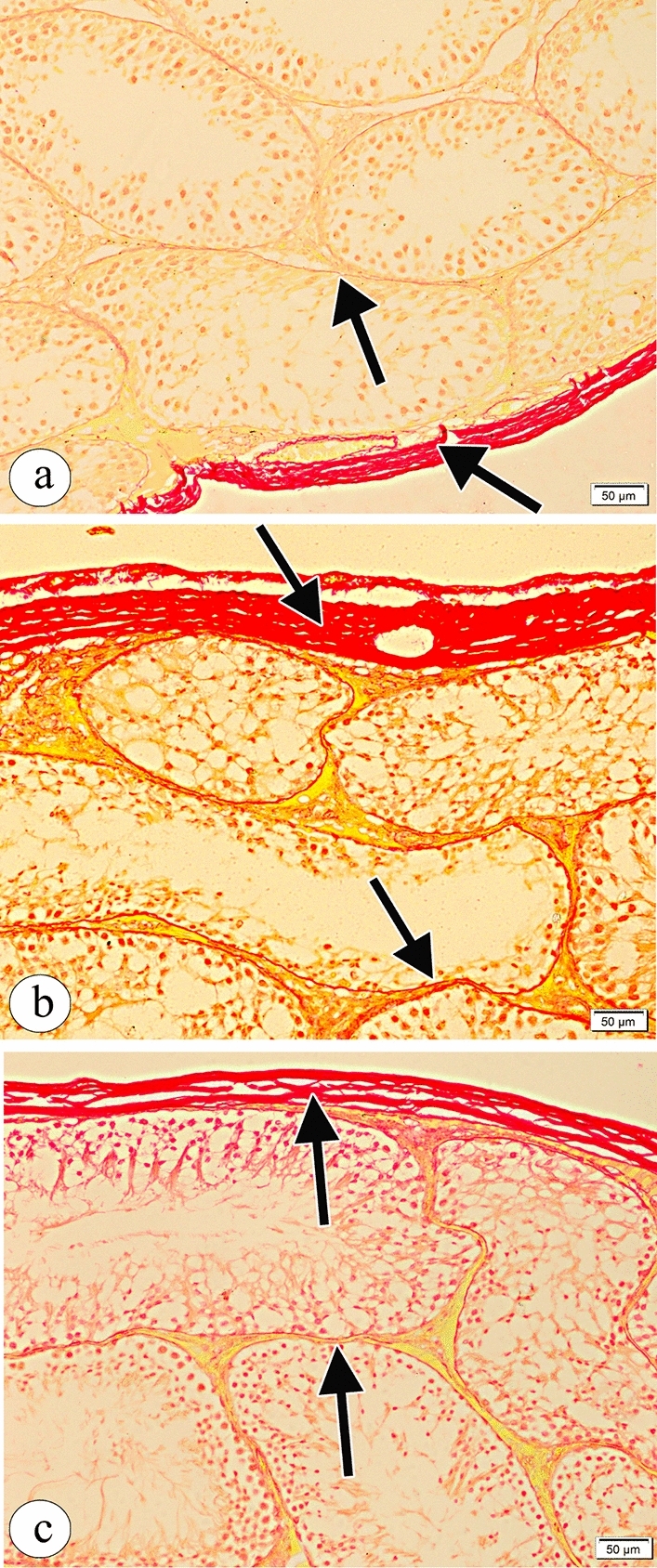
Photomicrographs of the sections of the testes in (**a**) the control group showing few collagen fibres (arrow) in tunica albuginea and the interstitium. (**b**) The atrazine-treated group showing many collagen fibres (arrow) in tunica albuginea and the interstitium. (**c**) The atrazine + resveratrol-treated group showing few collagen fibres (arrow) in tunica albuginea and the interstitium similar to the control group. (Picrosirius red × 200, Scale bar = 50 μm).

Evaluation of Picrosirius red-stained sections demonstrated few collagen fibres in tunica albuginea and the interstitium in the control group (Fig. 4a). While in the atrazine-treated group, many collagen fibres in tunica albuginea and the interstitium were detected (Fig. 4b). The atrazine + resveratrol treated group showed few collagen fibres in tunica albuginea and the interstitium similar to the control group (Fig. 4c). The mean area percent of collagen fibres showed a statistical difference between all groups (Table 4 and Fig. 4).

**Table 4:** The comparison of the area percent (area %) of collagen deposition, the area percent (area %) of caspase expression and area percent (area %) of iNOS expression.

Groups	Area % of collagen deposition	Area % of caspase expression	Area % of iNOS expression
Group I	4.99 ± 0.47	3.88 ± 0.55	4.15 ± 0.77
Group II	4.05 ± 0.60	3.95 ± 0.56	3.85 ± 0.50
Group III	17.69 ± 0.79^a,b,d^	33.16 ± 1.61^a,b,d^	24.19 ± 1.42^a,b,d^
Group IV	10.95 ± 0.92^a,b,c^	4.56 ± 0.83 ^c^	14.67 ± 0.79^a,b,c^
*P*-value	< 0.0001*	< 0.0001*	< 0.0001*

Data are represented as Mean ± SD.* means statistically significant difference.

^a^statistically significant as compared with the group I, *P* < 0.05.

^b^statistically significant as compared with the group II, *P* < 0.05.

^c^statistically significant as compared with the group III, *P* < 0.05.

^d^statistically significant as compared with the group IV, *P* < 0.05.

### Effect of resveratrol on atrazine-induced ultrastructural damage

**Figure 5:**
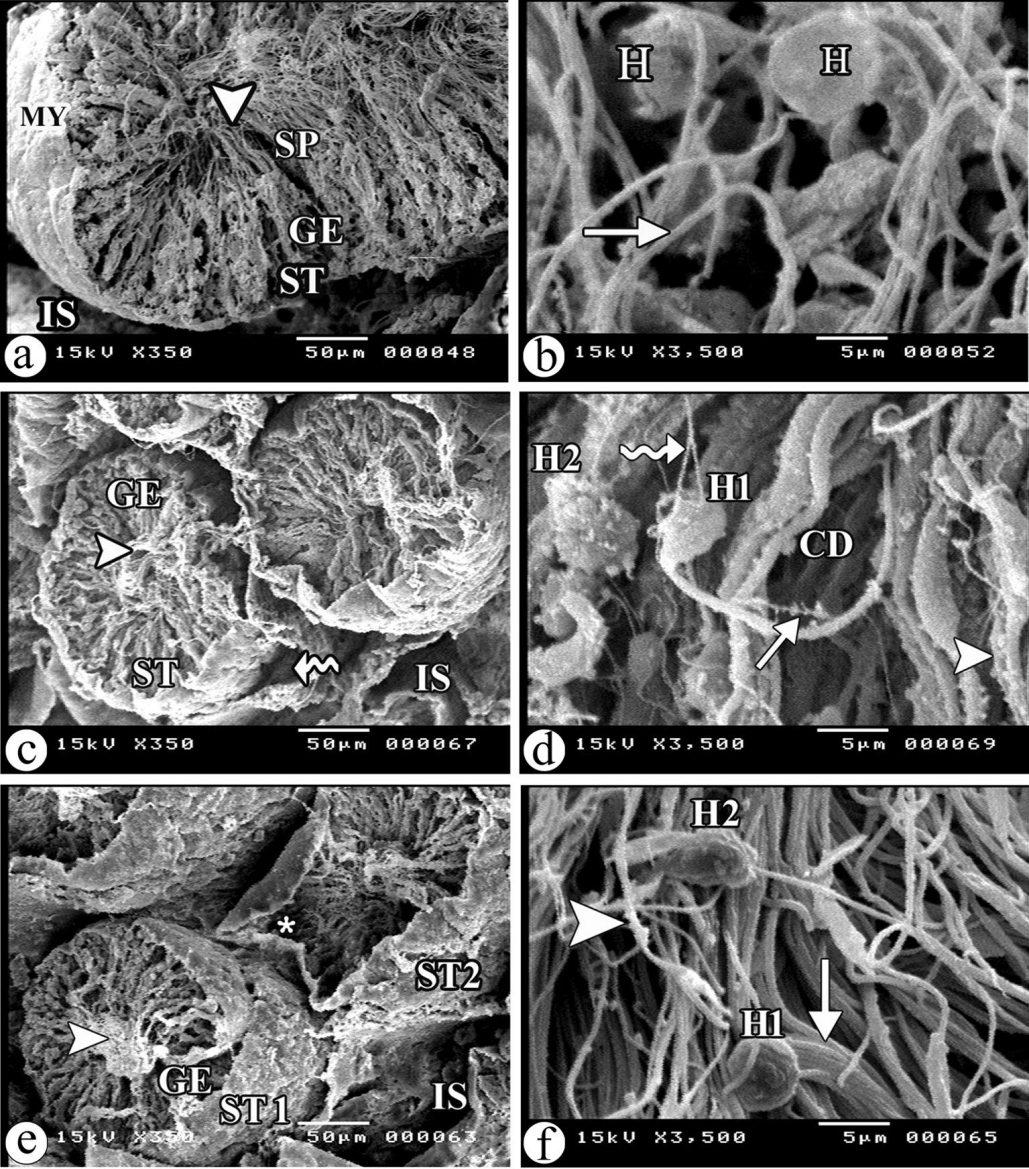
Scanning electron micrographs of the sections of testis in (**a**) the control group showing a seminiferous tubule (ST) with a regular outline, the germinal epithelium (GE), flat myoid cells (MY), many spermatids (SP) and long flagellae (arrowhead) of the spermatozoa in its lumen. Note interstitial spaces (IS). (**b**) The control group showing normal sperms with normal appearing heads (H) and tails (arrow). (**c**) The atrazine-treated group showing a shrunken seminiferous tubule (ST) with irregular outline, detachment (wavy arrow) of the germinal epithelium (GE) from the basement membrane, and few spermatozoa in its lumen (arrowhead). Note wide interstitial spaces (IS). (**d**) The atrazine-treated group showing a sperm has a head (H1) with detached part (wavy arrow) and tail splitting (arrow). Abnormal morphology of a head (H2) of another sperm is observed. Note the presence of membrane disruptions (arrowhead) and cytoplasmic droplets (CD) throughout the tails of many sperms. (**e**) The atrazine + resveratrol-treated group showing a shrunken seminiferous tubule (ST1) with a regular outline, the germinal epithelium (GE), and few spermatozoa in its lumen (arrowhead). Another shrunken seminiferous tubule (ST2) appears with an irregular outline, and vacuolations (asterisk). Note wide interstitial spaces (IS). (**f**) The atrazine + resveratrol-treated group showing a sperm with a normal appearing head (H1), and another sperm with abnormal appearing head (H2). Some tails of sperms appear normal (arrow), and others appear with membrane disruption (arrowhead). (SEM, a, c, e X350, scale bar = 5o μm and b, d, f X 3500, scale bar = 5 μm).

Scanning electron microscopic examination demonstrated the normal structure of the seminiferous tubules with many long flagella of the spermatozoa in their lumen in the control group (Fig. 5a). However, rats treated with Atrazine alone exhibited shrunken seminiferous tubules with irregular outlines, separation of the germinal epithelium from the basement membrane, and few spermatozoa in the lumen (Fig. 5c). The atrazine + resveratrol-treated group showed shrunken seminiferous tubules with regular outlines, and few spermatozoa in their lumens. Other shrunken seminiferous tubules appeared with irregular outlines, and vacuolations (Fig. 5e).

**Figure 6:**
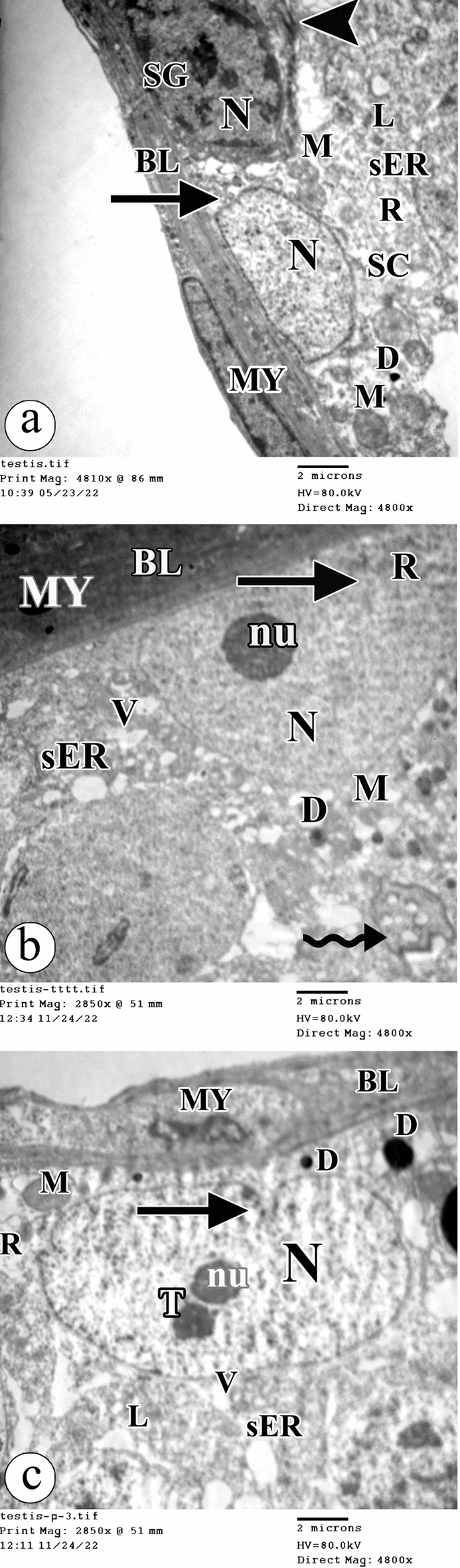
Electron photomicrographs of the ultra-thin sections of the testis in (**a**) the control group showing a spermatogonia cell (SG) having a dense nucleus (N) with condensation of the chromatin on the periphery and prominent nucleolus (nu). Note the presence of the tight junction (arrowhead) between adjacent cells. Sertoli cell (SC) is positioned on the normal basal lamina (BL) exhibiting myoid cell flat nucleus (MY). Sertoli cell have flat euchromatic nucleus (N) with characteristic invasion seen in the nuclear envelope (arrow). The cytoplasm shows smooth endoplasmic reticulum (sER), free ribosomes (R), many normal mitochondria (M), lysosomes (L) and lipid droplets (D). (**b**) The atrazine-treated group showing Sertoli cell is positioned on thickened basal lamina (BL) exhibiting myoid cell deeply stained nucleus (MY). Sertoli cell have flat dense nucleus (N) with a prominent nucleolus (nu) and intended nuclear envelope (arrow). The cytoplasm shows dilated smooth endoplasmic reticulum (sER), free ribosomes (R), vacuolations (V), crescent mitochondria (M), and lipid droplets (D). Note the presence of multivesicular body (wavy arrow). (**c**) The atrazine + resveratrol-treated group showing a Sertoli cell is positioned on the basal lamina (BL) exhibiting myoid cell (MY) with flat nucleus. A Sertoli cell has flat euchromatic nucleus (N) with a prominent nucleolus (nu), a satellite nucleolus (T) and characteristic invasion seen in the nuclear envelope (arrow). The cytoplasm shows dilated smooth endoplasmic reticulum (sER), free ribosomes (R), vacuolations (V), few normal mitochondria (M), lysosomes (L) and lipid droplets (D). (TEM, × 4800, scale bar = 2 μm).

Transmission electron microscopic examination demonstrated that the control group showed spermatogonia cells having dense nuclei with condensation of the chromatin on the periphery and prominent nucleoli. The presence of tight junctions was observed between adjacent cells. Sertoli cells were positioned on the normal basal lamina exhibiting myoid cells with flat nuclei. Sertoli cells had flat euchromatic nuclei with characteristic invasions seen in the nuclear envelopes. The cytoplasm showed smooth endoplasmic reticulum, free ribosomes, many normal mitochondria, lysosomes, and lipid droplets (Fig. 6a). The atrazine-treated group had Sertoli cells positioned on thickened basal lamina exhibiting myoid cells with deeply stained nuclei. Sertoli cells had flat dense nuclei with prominent nucleoli and intended nuclear envelopes. The cytoplasm showed dilated smooth endoplasmic reticulum, free ribosomes, vacuolations, crescent mitochondria, and lipid droplets. Additionally, the presence of multivesicular bodies was observed (Fig. 6b). The atrazine + resveratrol treated group showed Sertoli cells positioned on the basal lamina exhibiting myoid cells with flat nuclei. Sertoli cells had flat euchromatic nuclei with prominent nucleoli, satellite nucleoli, and characteristic invasions seen in the nuclear envelopes. The cytoplasm revealed dilated smooth endoplasmic reticulum, free ribosomes, vacuolations, a few normal mitochondria, lysosomes, and lipid droplets (Fig. 6c).

**Figure 7:**
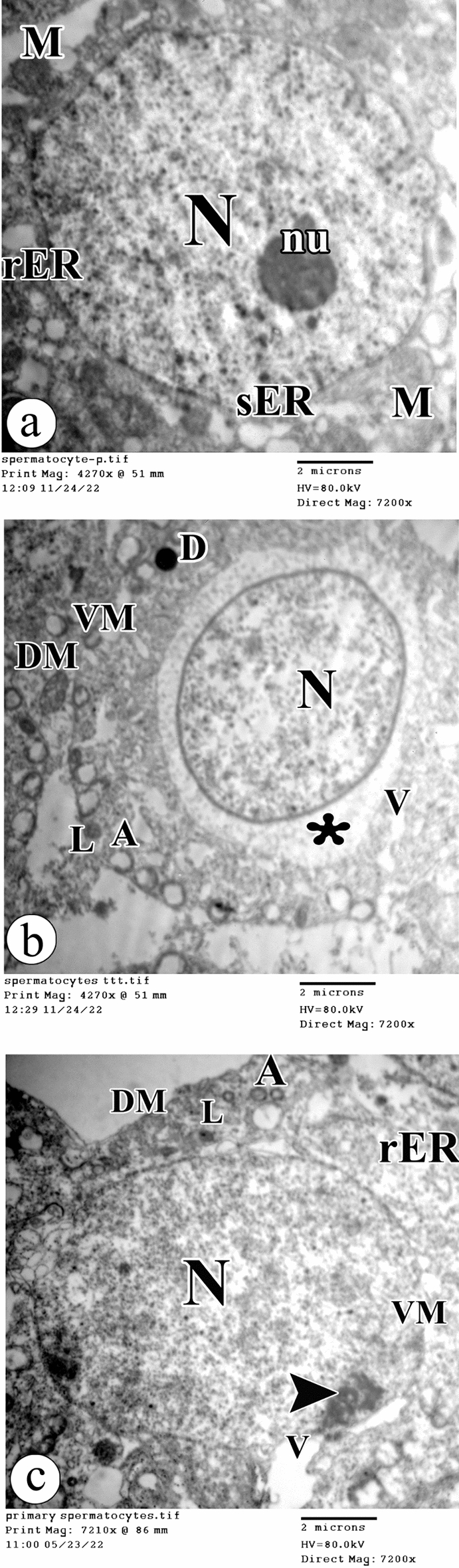
Electron photomicrographs of the ultra-thin sections of the testis in (**a**) the control group showing a primary spermatocyte has an euchromatic nucleus (N) and prominent nucleolus (nu). The cytoplasm shows smooth endoplasmic reticulum (sER), rough endoplasmic reticulum (rER), and the peripherally positioned mitochondria (M). (**b**) The atrazine-treated group showing a primary spermatocyte has a shrunken euchromatic nucleus (N). The cytoplasm shows an area of rarefication (asterisk), vacuolations (V), destructed mitochondria (DM), vacuolated mitochondria (VM), autophagic vacuoles (A), lysosomes (L), and lipid droplets (D). (**c**) The atrazine + resveratrol-treated group showing a primary spermatocyte has a nucleus (N) with clumped chromatin (arrowhead). The cytoplasm shows rough endoplasmic reticulum (rER), destructed mitochondria (DM), vacuolated mitochondria (VM), autophagic vacuoles (A), and lysosomes (L). (TEM, × 7200, scale bar = 2 μm).

Regarding the primary spermatocytes, they appeared with euchromatic nuclei and prominent nucleoli in the control group. The cytoplasm showed smooth endoplasmic reticulum, rough endoplasmic reticulum, and peripherally positioned mitochondria (Fig. 7a). However, the primary spermatocytes had shrunken nuclei in the atrazine-treated rats. The cytoplasm revealed areas of rarefication, vacuolations, destructed mitochondria, vacuolated mitochondria, autophagic vacuoles, lysosomes, and lipid droplets (Fig. 7b).

The primary spermatocytes had nuclei with clumped chromatin in rats exposed to atrazine and resveratrol. The cytoplasm contained rough endoplasmic reticulum, destructed mitochondria, vacuolated mitochondria, autophagic vacuoles, and lysosomes (Fig. 7c).

**Figure 8:**
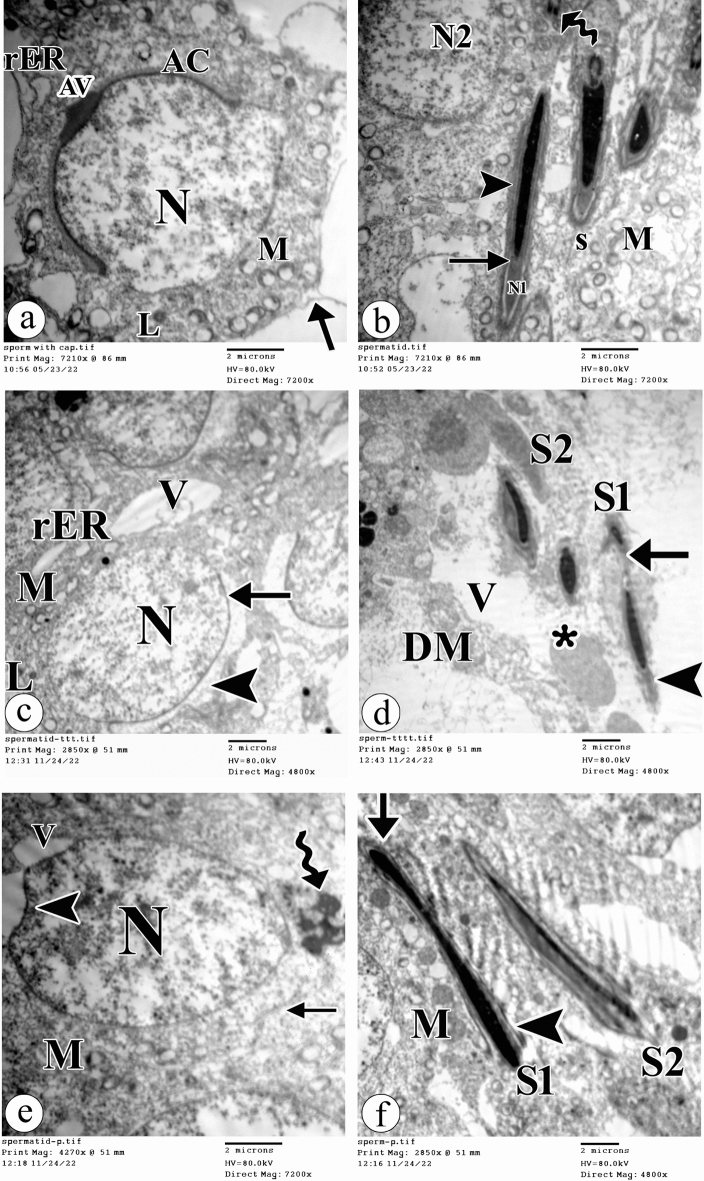
Electron photomicrographs of the ultra-thin sections of the testis in (**a**) the control group showing a rounded spermatid has a rounded euchromatic nucleus (N) partially covered on one side of its circumference with acrosomal cap (AC) and acrosomal vesicle (AV). The cytoplasm shows mitochondria (M), rough endoplasmic reticulum (rER), and lysosomes (L). The caudal end of the nucleus is encircled by an incomplete sheath made of microtubules (arrow). (**b**) The control group showing an elongated spermatid (mature spermatozoon) has a pyriform-shaped nucleus (N1) with condensed chromatin, the middle piece has central microtubules (arrow) and a well-formed tail (arrowhead). Numerous peripheral mitochondria (M) with a clear matrix and unclear cristae are present in the cytoplasm. Notice a circular euchromatic nucleus (N2) of rounded spermatid, different stages of maturity of sperms (S), and stages of spermiogenesis (wavy arrow). (**c**) The atrazine-treated group showing a rounded spermatid has a rounded euchromatic nucleus (N) with incomplete loss of acrosomal cap (arrow). The cytoplasm shows few mitochondria (M) with a clear matrix, dilated rough endoplasmic reticulum (rER), and lysosomes (L). Note the presence of several vacuoles (V) and large lysosome (acrosin) (arrowhead) covering the anterior part of spermatid. (**d**) The atrazine-treated group showing a spermatozoon (S1) has a degenerated head (arrowhead) and broken tail (arrow). The surrounding cytoplasm shows destructed mitochondria (DM) and numerous vacuoles (V). Note the abnormal shape of another spermatozoon (S2), and lamellated bodies (asterisk). (**e**) The atrazine + resveratrol-treated group showing a rounded spermatid has an euchromatic nucleus (N) with irregular nuclear membrane (arrowhead), loss of the acrosomal cap, vacuolation (V), and smashed centrioles at the other side of the nucleus (wavy arrow). The caudal end of the nucleus is encircled by an incomplete sheath made of microtubules (arrow). (**f**) The atrazine + resveratrol-treated group showing one spermatozoon (S1) appears normal with head (arrow) and tail (arrowhead). Another spermatozoon (S2) appears deformed. Numerous normal mitochondria (M) are present in the cytoplasm. (TEM, × 7200, scale bar = 2 μm).

Regarding the rounded spermatid, in the control group, it had a rounded euchromatic nucleus partially covered on one side of its circumference with an acrosomal cap and acrosomal vesicle. The cytoplasm contained many mitochondria, rough endoplasmic reticulum, and lysosomes. The caudal end of the nucleus was encircled by an incomplete sheath made of microtubules (Fig. 8a). With atrazine treatment, the rounded spermatids had rounded euchromatic nuclei with incomplete loss of acrosomal caps. The cytoplasm contained few mitochondria with a clear matrix, dilated rough endoplasmic reticulum, and lysosomes. Several vacuoles and large lysosome (acrosin) covering the anterior part of the spermatid were detected (Fig. 8c). In the atrazine + resveratrol treated group, the rounded spermatid was still affected and had a euchromatic nucleus with irregular nuclear membrane, loss of the acrosomal cap, vacuolation, and smashed centrioles at the other side of the nucleus. The caudal end of the nucleus was encircled by an incomplete sheath made of microtubules (Fig. 8e). Table 3 displays the histopathological scoring observed in the different groups.

### Effect of resveratrol and atrazine on the sperm morphology

Scanning electron microscopy was used to observe sperm morphology. The control group showed normal sperms with normal morphology of the heads and tails (Fig. 5b). The atrazine-treated group showed some sperms with detached parts of the heads and tail splitting, abnormal morphology of the heads of others, and the presence of membrane disruptions as well as cytoplasmic droplets throughout the tail of many sperms (Fig. 5d). The atrazine + resveratrol treated group showed little improvement in the sperm morphology. Some sperms with normal-appearing heads and others with abnormal morphology of the heads were observed. Also, some tails of sperms appeared normal, but others revealed membrane disruptions (Fig. 5f).

In addition, a transmission electron microscopic study was performed to observe sperm morphology. In the control group, the elongated spermatids (mature spermatozoa) were formed of pyriform-shaped nuclei with condensed chromatin, the middle pieces with central microtubules and well-formed tails. Numerous peripheral mitochondria with clear matrix and unclear cristae were present in the surrounding cytoplasm. Additionally, circular euchromatic nuclei of rounded spermatids, different stages of maturity of sperms, and stages of spermiogenesis were seen (Fig. 8b). In the atrazine-treated group, the spermatozoa had degenerated heads and broken tails. The surrounding cytoplasm showed destructed mitochondria and numerous vacuoles. Abnormal shapes of spermatozoa and lamellated bodies were observed (Fig. 8d). The atrazine + resveratrol treated group showed the spermatozoa appeared normal. Other spermatozoa appeared deformed. Numerous normal mitochondria were detected in the surrounding cytoplasm (Fig. 8f). Table 3 displays the histopathological scoring observed in the different groups.

### Effect of resveratrol administration on the immunohistochemical reaction in the testes of the rats exposed to atrazine

**Figure 9:**
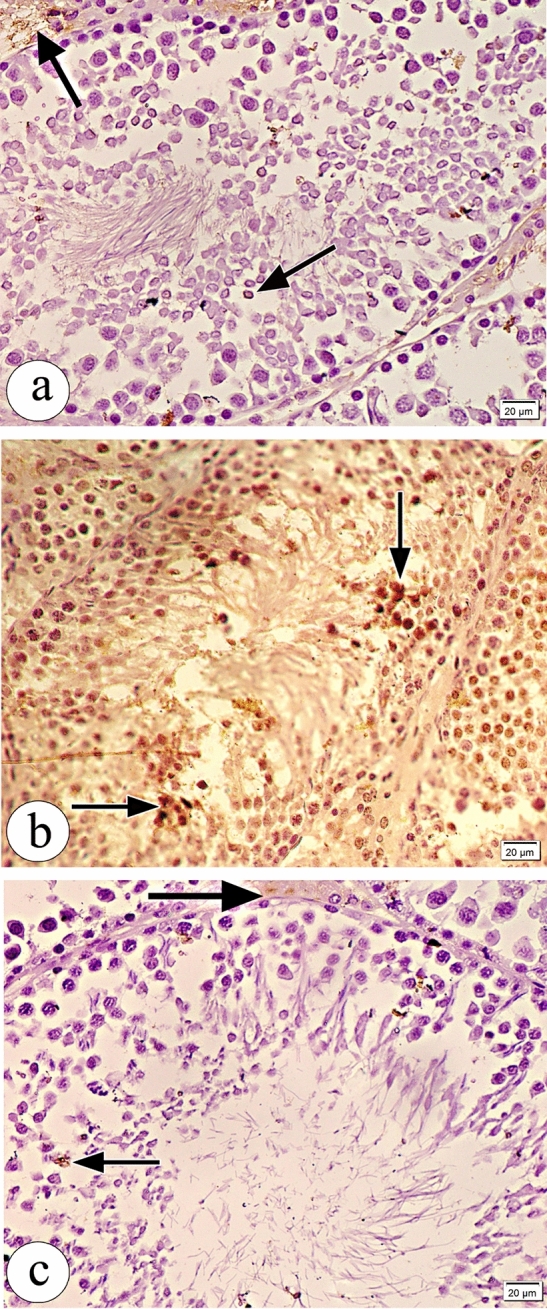
Photomicrographs of the sections of the testes in (**a**) the control group showing weak positive caspase immunoreaction (arrow). (**b**) The atrazine-treated group showing strong positive caspase immunoreaction (arrow). (**c**) The atrazine + resveratrol-treated group showing weak positive caspase immunoreaction (arrow). (caspase counterstained with haematoxylin, × 400, scale bar = 20 μm).

**Figure 10:**
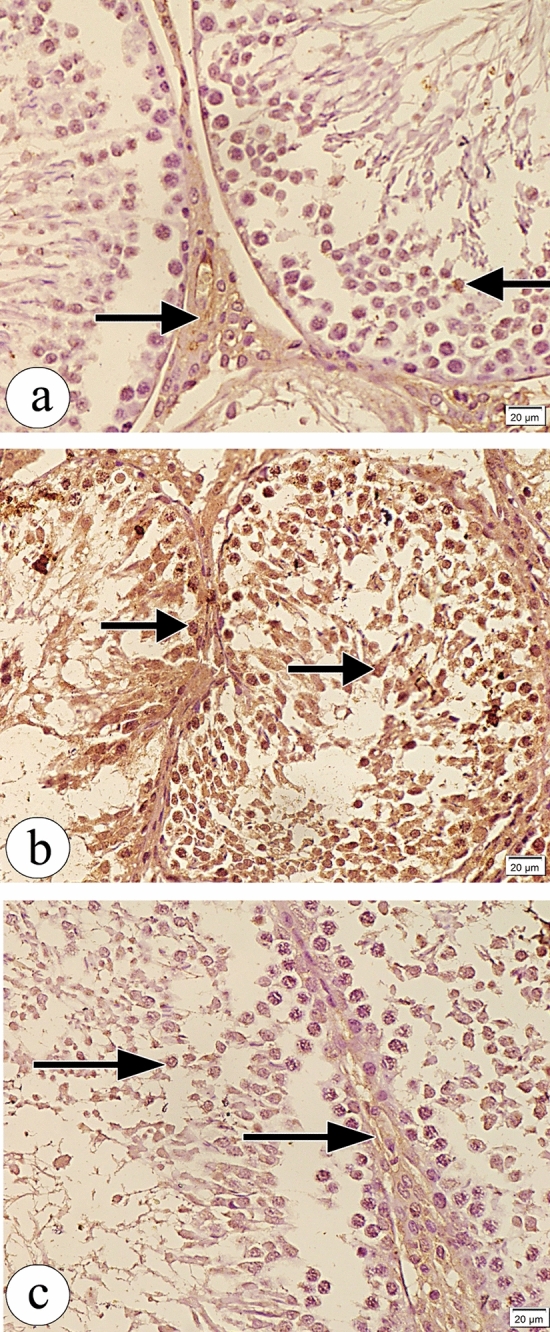
Photomicrographs of the sections of testes in (**a**) the control group showing weak positive iNOS immunoreaction (arrow). (**b**) The atrazine-treated group showing strong extensive positive iNOS immunoreaction (arrow). (**c**) The atrazine + resveratrol-treated group showing weak positive iNOS immunoreaction (arrow). (iNOS counterstained with haematoxylin, × 400, scale bar = 20 μm).

Examination of immunohistochemical stained testicular sections obtained from the control group revealed weak positive caspase immunoreaction and weak positive iNOS immunoreaction (Figs. 9a and 10a respectively), whereas those obtained from the atrazine-treated group revealed strong positive caspase immunoreaction and strong extensive positive iNOS immunoreaction (Figs. 9b and 10b respectively). The atrazine + resveratrol treated group revealed weak positive caspase immunoreaction and weak positive iNOS immunoreaction (Figs. 9c and 10c respectively). There were statistically significant differences in the percent of expression area of caspase and iNOS between the studied groups (Table 4).

### Effect of resveratrol and atrazine on the height of germinal epithelium, and the area of seminiferous tubules

The morphometric and statistical studies demonstrated that the atrazine-treated group showed a significant decrease in the height of germinal epithelium, and the area of seminiferous tubules when compared to the control group (Table 4). On the contrary, the atrazine + resveratrol-treated group revealed a significant increase in the prior parameters when compared to the atrazine-treated group (Table 5).

**Table 5:** The comparison of the height of the germinal epithelium (µm), and the area of the seminiferous tubules (µm^2^).

Groups	Height of germinal epithelium (µm)	Area of seminiferous tubules (µm ^2^)
Group I	178.6 ± 12.87	459,250.43 ± 145,405
Group II	184.4 ± 8.76	349,358.18 ± 78,086
Group III	98.64 ± 15.32^a,b,d^	56,083.03 ± 18741^a,b^
Group IV	129.3 ± 15.03^a,b,c^	161,143.56 ± 62503^a,b^
*P*-value	< 0.0001*	< 0.0001*

Data are represented as Mean ± SD.* means statistically significant difference.

^a^statistically significant as compared with the group I, *P* < 0.05.

^b^statistically significant as compared with the group II, *P* < 0.05.

^c^statistically significant as compared with the group III, *P* < 0.05.

^d^statistically significant as compared with the group IV, P < 0.05.

## Discussion

One type of herbicide, atrazine, is frequently used to weed dry crops including sorghum, fruit trees, and corn. It is simple to continuously pollute the environment because of its lengthy half-life in soil and resistant breakdown in water. In dairy products like milk and yoghurt, atrazine has also been discovered. Atrazine may enter the body of a human by food or water, and other methods, harming several organs^1^.

Several studies concluded that atrazine-induced degenerative changes in the liver^14,40^, kidney^41^, the hippocampus^42^, the striatum^43^, the cerebellum and thyroid gland^44^.

Atrazine is a potent endocrine disruptor that may have negative consequences on reproduction in several vertebrate species, including amphibians, fish, birds, amphibians, and mammals^45^.

In the rat seminiferous tubule culture model, exposure to atrazine reduced the number of spermatids by disrupting the blood-testis barrier's claudin-11 and connexin-43 proteins and affecting spermatocytes^46^.

Male fertility is negatively impacted by atrazine exposure. This proves that precautions must be taken to avoid atrazine exposure's damaging effects on male fertility^45^.

An increasing volume of data confirmed the adverse effects of atrazine on the testis^17,45,47,48^, the aim of the present work was to assess the potential protective role of resveratrol against the adverse effects of atrazine. To our knowledge, this work is the first study investigated the protective impact of resveratrol against testicular damage resulting from atrazine exposure in male albino rats.

In the current study, the selected atrazine dose was chosen as the lowest sublethal dose (50 mg/kg) used in many studies. It is estimated about 1/60 of the LD50. When given orally to rats, atrazine has a lethal dose of 3090 mg/kg as calculated by Extension Toxicology Network in the United States^49^. In addition, the selected resveratrol dose (20 mg/kg bw) was chosen in the current work as it more or less is found to be protective against testicular toxicity induced by many drugs in many studies^29,50^. It was documented that resveratrol has positive impacts on the reproductive systems of both male and female rats^51^.

People are exposed to atrazine in many ways through breathing, drinking, eating, and touching from contaminated drinking water and food, direct contact with farm workers and herbicide applicators, digging in dirt containing atrazine in it, and children playing in dirt containing^52^. The potential health hazards for the exposed persons including cancer, congestion of the heart, lungs, and kidneys, low blood pressure, muscle spasms and degeneration, weight loss, as well as degeneration of adrenal glands, cardiovascular system, and retina^53^.

Swan et al. (2003) conducted the first investigation to investigate the dangers to human reproduction associated with exposure to atrazine. This population-based study showed the relationship between environmental exposure biomarkers and human male reproduction, and it concluded that pesticide use was associated with lower-than-expected semen quality, indicating that atrazine may have contributed to lower-than-expected semen quality in fertile men^54^.

A previous study found that atrazine may activate the examined steroidogenesis genes in interstitial Leydig cells through a mechanism that is not dependent on cyclic AMP^55^.

Prior authors investigated the effects of long-term atrazine (5 mg/kg bw/day) exposure in drinking water on male mice's metabolism and reproductive characteristics from E9.5 to 12 or 26 weeks of age. They found that atrazine reduced the weight of the liver, reduced the concentration of epididymal sperm, and altered the expression of genes in the liver and testes, particularly those related to the liver's fatty acid metabolism and lipid uptake, and the testis' androgen conversion^56^. Also, the percentage of dead spermatozoa significantly increased and reduced motility of epididymal sperm in mice given a low dose of atrazine from weaning for eight weeks, along with an increase in weight gain overall and cumulatively^57^.

The current research found that atrazine exposure at a daily dose of 50 mg/kg for one month led to a significant decrease in serum testosterone hormone level, upregulation of caspase 3 and iNOS mRNA levels, destructed seminiferous tubules with few sperms in their lumens, damaged germinal epithelium separating it from the basement membranes, dilated congested blood vessels, few Leydig cells, acidophilic material deposition in wide vacuolated interstitial spaces, the irregular outline of some nuclei of primary spermatocytes, shrunken nuclei of other primary spermatocytes, loss of acrosomal caps of round spermatids, ill-defined germ cells, some germ cells shed and sloughed to the lumen, and many collagen fibres accumulation in the tunica albuginea and the interstitium. The cytoplasm of many germ cells had dilated smooth endoplasmic reticulum, vacuolations, damaged mitochondria, and vacuolated mitochondria. Additionally, abnormal morphology of some sperms in the form of detached parts of the heads, tail splitting, as well as the presence of membrane disruptions and cytoplasmic droplets throughout the tail of many sperms were detected. Moreover, strong positive caspase immunoreaction and strong extensive positive iNOS immunoreaction in the testicular sections were noticed. Concomitant administration of resveratrol can improve these adverse effects.

The ability of male rats given atrazine to fertilize was significantly decreased compared to the control rats. This may be linked to the decline in serum testosterone levels, impaired spermatogenesis and sperm maturation events, or both. It was documented that testosterone has been linked to enhanced sexuality, greater vigor, stamina, and mental and physical energy^58^.

The present data for serum testosterone levels agreed with those from previous studies^17,47,48^. In addition, previous data showed that exposure to atrazine during pregnancy can cause hypospadias in mice, probably by disrupting the generation of testosterone, lowering the effect of androgens, and subsequently changing the expression of sexually differentiating genes^59^.

On the other hand, the results of early work concluded that atrazine treatment of up to 50 mg/kg per day induced no effect on the serum testosterone level and hypothalamic-pituitary–testicular axis^47^.

The production of testosterone and the action of pituitary gonadotropins like luteinizing hormone (LH), which stimulates the creation and release of testosterone by Leydig cells, and follicle-stimulating hormone (FSH), which promotes testicular growth and increases the production of an androgen-binding protein by Sertoli cells, are the two main factors that influence the spermatogenesis process^60^.

It was suggested that the low serum testosterone level in this study may be attributable to the function of atrazine as an endocrine-disrupting substance having effects on the endocrine system^61^. Additionally, Leydig cells which is the principal site of the androgen synthesis in the testes, are directly harmed by atrazine^62^. Therefore, it could be suggested that a rat’s reproductive system may be harmed by atrazine since testosterone is necessary for the growth of the sex organs as well as the productivity of sperm and resveratrol can reverse this effect. Also, we suggested that the present observed changes in testosterone levels were linked to the testis's histological abnormalities.

The present findings were in the same line with an early study of investigators who found that the gonadal hormones and all rat testicular function indicators dramatically declined in the atrazine-treated group, associated with histoarchitecture deterioration and abnormal sperm characteristics^45^. Similarly^63^, found that the mice given atrazine displayed a markedly reduced body weight and testis weight, loosely arranged seminiferous tubules, disarrangement, and fewer spermatogenic cells, as well as decreased sperm concentration.

Interstitial vacuolations were clearly observed in this study, which may be connected to the released steroid hormone produced by Leydig cells^2^. Additionally, acidophilic material deposition and dilated blood vessels in the interstitium were also noted in this work. In this context, some researchers claimed that the chemical mediators produced soon after tissue damage enhance the permeability of blood vessel walls, causing plasma to leak into the surrounding tissue and subsequently forming the fluid exudate and acidophilic material deposition^64^.

Sertoli cells in the current study showed major affection with atrazine exposure in the form of dense nuclei, dilated smooth endoplasmic reticulum, cytoplasmic vacuolations, crescent mitochondria, and vacuolated mitochondria. We have suggested that there is a link between atrazine exposure and the disintegration of Leydig and Sertoli cell’s function. Sertoli cells form the blood-testis barrier and are regarded as the metabolic depots that provide sufficient amounts of energy substrates and growth factors to germ cells^65^. Additionally, Sertoli cells promote spermatogenesis, hence it is commonly believed that the interactions between Sertoli cells and germ cells regulate how long cell cycles last and how cells are organized^66^.

The present cytoarchitecture damage in the spermatocytes and spermatids is an indication of impeding the spermatogenesis process. These findings may be linked to disruptions in the Sertoli cells' microenvironment that have an impact on the machinery needed for protein production and differentiation of the germ cells^67^. According to a previous work, the first Sertoli cell damage causes germ cells apoptosis and the production of reactive oxygen species (ROS), which can change the integrity of the sperm cell membranes and cause sperm to lose their motility^68^. Moreover, certain research has linked the formation of lipid droplets and multivesicular bodies in Sertoli cells to a rise in the phagocytic ability of apoptotic spermatogenic cells, which is consistent with our findings^69^ .

Seminiferous epithelial vacuoles in the present work have been linked to the loss of germ cells, whereas vacuolization in the cytoplasm of Sertoli cells is caused by aberrant germ cells that are phagocytized by Sertoli cells. This was in the same line with^70^.

The present work found that there was abnormal morphology of some sperms with exposure to atrazine. Similar to our findings, previous authors found that oral administration of atrazine (50 mg/kg b.w) for 6 days led to a decrease in sperm quality and an increase in the number of abnormal sperms^71^.

On the same line, early researchers found that the mice given atrazine at 100 mg/kg/d for four weeks displayed an increase in the percentage of morphologically abnormal sperm in the tail of epididymis^63^. Moreover, it has been observed that men in agricultural areas are less fertile and have lower-quality semen because of atrazine exposure^72^. On the other hand, another study reported that the testicular sperm number, sperm viability, and sperm production per day showed no change in atrazine-treated rats^15^. It is worthy of note that by evaluating sperm concentration, morphology, and motility, the fertility function of the male reproductive system can be assessed. In addition, for spermatogenesis to occur, the testosterone levels must be present^73^.

It was known that the final stage of spermatogenesis, known as spermiogenesis, is a highly coordinated developmental process. Spermiogenesis is the post-meiotic stage of male germ cell development in which the haploid round spermatid, just before its discharge into the lumen of the seminiferous tubule, differentiates into an elongating mature spermatozoon in the seminiferous epithelium^74^. Acrosome creation, chromatin content condensation, restructuring of the mitochondria, elimination of superfluous cytoplasm and organelles, shape remodeling of the nucleus, and sperm tail development are the main events of this phase^65^. Later on, in the spermiogenesis process, loss of extra spermatid material (cytoplasm, water, and organelles) which is not required for the spermatozoon was done via phagocytosis by the Sertoli cells^75^. Accordingly, the authors suggested that atrazine can impede spermatogenesis and resveratrol can prevent this.

To find the mechanism of atrazine-induced testicular damage, mRNA level expression assay, and immunohistochemical study were done. Atrazine exposure was found to induce apoptosis. This has been improved in the atrazine + resveratrol-treated group. These results were also consistent with previous research that found that the mice given atrazine at 100 mg/kg/d for four weeks displayed increased spermatocyte apoptosis rate^63^. It is known that caspases are cysteine proteases and are known as one of the pro-apoptotic proteins that encourage mammalian apoptosis. Caspase-3 is activated by death receptors to cause apoptosis^76^. It was discovered that the cleavage of gasdermin E (GSDME), a protein, by caspase-3 in reaction to several kinds of cellular stress, results in a fragment that can pierce the cell membrane and cause the cell to burst and spill its contents. It is believed that a key mechanism for eradicating injured or infected cells is the caspase-3/GSDME signaling pathway^77^.

Apoptosis is thought to be indicated by the phosphatidylserine leaving the cell and moving to the sperm's and germ cell’s outer membrane, the activation of caspases, and chromosome fragmentation^78^. Apoptosis is a unique mechanism that causes DNA dispersion and is characterized by chromatin breakage during planned cell death^79^.

We hypothesize that the apoptotic rate observed in this study was pathological, even though testicular germ cell apoptosis is thought to be a physiological mechanism to reduce the germ cell population to a number that can be nursed by Sertoli cells^80^. This is because the testis of atrazine-exposed rats showed significantly higher expression of cleaved caspase-3 than did the control rats. It was suggested that the apoptotic signaling pathway is activated as a result of the vicious cycle of stimuli created by the interaction between oxidative damage and inflammation^81^.

From the earliest embryonic stages of gonadal differentiation to fertilization, apoptosis controls the male germ cells' proper development and function. Also, apoptosis is among the commonly recognized regulatory processes in the testis, and it acts to maintain a proper ratio between Sertoli and germ cells^68^.

Although to a lesser extent, apoptosis also takes place during the maturation and divisions of spermatocytes and spermatids^82^. However, an increase in apoptosis can be harmful to sperm production^68^. In addition, DNA dispersion is a typical outcome of damage caused by ROS, and it's frequently noticed in spermatozoa from infertile men^83^. High ROS levels led to an accelerated and improperly controlled apoptotic response. Moreover, the various types of male infertility are greatly influenced by ROS^68^.

The second suggested mechanism underlying present atrazine-induced testicular toxicity is its ability to induce inflammation which is indicated by up-regulation of iNOS mRNA levels in the rats exposed to atrazine. Inducible nitric oxide synthase (iNOS) is responsible for the generation of reactive nitrogen species such as nitric oxide (NO), which one of the proinflammatory agents. The overproduction of NO causes inflammation^84^. While uncontrolled inflammatory reactions can result in severe and permanent tissue damage, inflammatory illnesses, and testicular pathogeneses, inflammation is the body's protective mechanism in the event of tissue injury^85^.

Collectively, testicular damage induced by atrazine exposure could be explained by several reasons. Atrazine is an environmental endocrine disruptor that can harm the endocrine-nervous system and associated endocrine axes, including the hypothalamic–pituitary–adrenal axis and the hypothalamus–pituitary–gonadal axis by breaching the blood–brain barrier and adversely affect the endocrine organs including the testes^86^. By preventing the release of GnRH, atrazine reduces the output of LH and FSH which are produced by the anterior pituitary and consequently decrease testosterone production. In addition, via affection of the steroid synthesis, atrazine has negative effects on the reproductive system^10^.

According to Gomes-Andrade et al., 2024, when Sertoli cells were exposed to atrazine, their oxidoreductase activity reduced, which may indicate a drop in their metabolic rate. Also, atrazine markedly reduced both glycolysis and the glycolytic capacity of Sertoli cells through downregulation of the protein levels of lactate dehydrogenase, according to the evaluation of the glycolytic function. There may be a connection between atrazine exposure and male infertility since lactate is the preferred metabolic substrate for germ cells, and exposure to atrazine may negatively affect the nutritional support essential for spermatogenesis^87^.

Furthermore, compared to other tissues, the testes and also sperms are substantially more susceptible to peroxidation harm because they are high in polyunsaturated fatty acids and have weak antioxidant defences^88^.

In addition, the involvement of oxidative stress in the testis of atrazine-treated rats is supported by the decrease in testicular superoxide dismutase (SOD) activity, and elevation of malondialdehyde (MDA) levels. Elevated MDA have negative effects on spermatogenesis. Rat testicular maturation and spermatogenesis are significantly influenced by SOD. Consequently, growth arrest and compromised testicular function may result from any change in testicular SOD activity. Because of the reduced SOD activity, superoxide anions may continue to be produced, which could harm spermatozoa^89^.

By findings of previous study concluded that atrazine altered the expression of testicular 3-hydroxysteroid dehydrogenase (3-HSD protein), a crucial enzyme in the androgen biosynthesis pathway, so this could contribute to our understanding of the consequences of atrazine as an endocrine disruptor. 3-HSD enzyme is involved in the steroidogenic pathway at several stages, including the production of the precursor to testosterone, androstenedione. Because of this, the interference of atrazine with steroidogenesis causes the Leydig cell to produce less testosterone, which compromises the spermatogenic process and the preservation of secondary sexual functions. Atrazine also changed the amounts of testosterone and estradiol in the plasma and testis of adult male rats, which had an impact on the histological architecture of the testis^90^.

Moreover, other study on adult mice given atrazine (50 mg/ kg) by gavage for three days showed decreased levels of 3β-hydroxysteroid dehydrogenase positive Leydig cells (LCs), which were linked to higher levels of caspase-3 immunoexpression and in situ cell death fluorescence in the testes. As a result, increased expressions of p45 were demonstrated by immunostaining for cell cycle gene regulators, along with increased expressions of cyclin D2 and E2^91^. This were in consistency with our study, the authors concluded that through apoptosis, atrazine appears to directly reduce the number of LCs that secrete testosterone.

The present study demonstrated a protective impact of resveratrol co-treatment against atrazine exposure through decreasing the testicular fibrosis, the caspase 3, and iNOS mRNA levels as well as increasing the serum testosterone and improving the structure of the seminiferous tubes, germ cells, and sperms. The present findings were in the same line with the previous study which showed that resveratrol is beneficial for ameliorating methotrexate-induced acute testicular injury, oxidative stress, and apoptosis through biochemical, histochemical, and immunohistochemical analyses^92^.

Consuming resveratrol has been demonstrated to be effective in the protection of several tissues from oxidative damage brought on by harmful metals and chemicals found in the environment. Because of its antioxidant, antimicrobial, and anti-inflammatory qualities, resveratrol is being researched to treat a wide range of inflammatory disorders, cardiovascular diseases, and cancer^93^.

Reduction of ROS production, elevation of DNA content of mitochondria, membrane potential, and electron transport chain complex activities are possible ways that resveratrol administration can enhance mitochondrial structure and function^94^. Furthermore, a rat study revealed that resveratrol boosted the production of neuronal nitric oxide synthase (nNOS) and sirtuin-1, lowered the rate of cell death and promoted the differentiation of rat germ cells^95^. Despite resveratrol's ability to protect spermatozoa from oxidative damage, its exact mechanisms of action remain unknown.

### Limitations of the study

Fertility assessments, sperm quality analyses, isolation of Leydig cells, and investigation of the steroidogenic capacity and activity as well as investigation of gonadotropin levels will enhance the comprehensiveness of the effect of atrazine exposure on the testis, hypothalamus, and pituitary gland. In addition, repeat experiments using several environmentally relevant concentrations of atrazine will provide more meaningful insights into the effects of atrazine exposure.

## Conclusion and recommendation

According to the current data, atrazine exposure is toxic to the testis and affects the normal structure of the seminiferous tubules and sperms. Through alterations to testicular morphology, a rise in the apoptosis of the germ cells, and fibrosis, atrazine can decrease spermatogenesis and impair male fertility in adult rats. Coadministration of resveratrol guards against this toxicity via the modulation of testosterone hormone, caspase 3, and iNOS mRNA level, as well as histoarchitecture of testes and sperms. It was concluded that consuming resveratrol treatment may be a worthwhile protective agent strategy to prevent the reproductive problems brought on by exposure to atrazine. This study clearly shows that using pesticides requires precautions, and further research is required to fully comprehend the toxicity and safety considerations of herbicide formulations made and used in Egypt.

## Data Availability

Available from the corresponding author upon reasonable request.
